# Identification of a novel cancer-associated fibroblasts gene signature based on bioinformatics analysis to predict prognosis and therapeutic responses in breast cancer

**DOI:** 10.1016/j.heliyon.2024.e29216

**Published:** 2024-04-03

**Authors:** Jin Song, Huifeng Liao, Huayan Li, Hongye Chen, Huiyan Si, Jiandong Wang, Xue Bai

**Affiliations:** a Department of General Surgery, The First Medical Center of Chinese PLA General Hospital, Beijing, 100853, China; b Department of General Surgery, The Seventh Medical Center of Chinese PLA General Hospital, Beijing, 100700, China; c The Second School of Clinical Medicine, Southern Medical University, Guangzhou, 510515, China; d Department of Gynecology, Zhujiang Hospital, Southern Medical University, Guangzhou, 510280, China

**Keywords:** Cancer-associated fibroblasts, WGCNA, Breast cancer, Tumor microenvironment, Prognosis

## Abstract

Cancer-associated fibroblasts (CAFs) provide suitable conditions for growth of tumor cell and facilitate tumor progression. Hence, we aimed to identify a CAFs-related gene signature associated with the prognosis of patients with breast cancer (BRCA). We downloaded datasets from Gene Expression Omnibus (GEO) and confirmed the correlation between CAFs infiltration scores and prognosis. By performing weighted gene co-expression network analysis (WGCNA) and Lasso Cox regression analysis, we constructed a four-gene (COL5A3, FN1, POSTN, and RARRES2) prognostic CAFs signature model. Based on the median risk score of CAFs, patients with BRCA were divided into high- and low-risk groups. Compared with low-risk group, patients in high-risk group exhibited a poor prognosis and limited response to immunotherapy. Furthermore, patients with high CAFs risk scores were found to have a detrimental prognosis due to the induction of immunosuppressive cell infiltration, resulting in an immunosuppressive tumor microenvironment. Importantly, we found that CAFs overexpressing FN1 and POSTN significantly promoted the wound healing and invasion ability of tumor cells in vitro validation. Taking together, we identified a four-gene prognostic CAFs signature, which was proven to be a reliable indicator for prognosis and therapeutic efficacy in patients with BRCA. This study provided evidence for novel CAFs-based stromal therapy.

## 1 Introduction

The latest global cancer statistics indicate BRCA is currently the most prevalent cancer among women, ranking fifth in cancer-related mortality. Its incidence rate is increasing rapidly across continents such as South America, Africa, and Asia [1]. In 2020, 20000 new cases and 685000 deaths were reported worldwide. The lack of effective BRCA treatment remains a major public health concern globally, greatly impacting the survival and quality of life for women. Despite advancements in chemotherapy, targeted therapy, immunotherapy, recurrence and metastasis caused by drug resistance are the primary causes of death in patients [2,3].

Tumor microenvironment (TME) comprises surrounding blood vessels, immune cells, fibroblasts, chemokines, and the extracellular matrix (ECM), and the intricate interactions between tumor cells and TME affect tumor growth, metastasis, and immunosuppression [4]. A deeper understanding and attention to the relevant cellular and molecular components of TME are conducive to the discovery of new therapeutic targets [5,6]. Among the most significant components of the TME, CAFs affect tumor cell proliferation, migration, invasion, treatment resistance, and distant metastasis [[7], [8], [9], [10]]. Many recent studies have shown that CAFs can promote tumor progression, ECM remodeling, inflammation, and immunosuppression [11,12]. For example, platelet-derived growth factors secreted by CAFs can activate and induce fibroblast proliferation, leading to tumor progression [13]. Additionally, CAFs-mediated immune regulation and angiogenesis may support cancer cell survival and facilitate evasion from the immune system [[14], [15], [16]]. Despite its crucial role in tumor development, there are currently no reliable prognostic markers for CAFs to predict BRCA prognosis and treatment responses. Thus, exploring reliable prognostic markers for CAFs is critical for formulating and optimizing treatment strategies.

WGCNA is a bioinformatics tool used to analyze gene expression patterns over multiple samples. WGCNA can identify associations between modules and specific traits or phenotypes by clustering genes with similar expression patterns [17]. This powerful tool is widely used in research on phenotypic traits and gene association analysis. WGCNA has been successfully applied to identify CAFs markers in gastric and ovarian cancers [[18], [19], [20]]. However, WGCNA has not been applied to CAFs in BRCA yet. Here, we determined a new prognostic biomarker for CAFs, which can serve as a powerful prognostic indicator for BRCA, thus helping recommend appropriate clinical treatment. Additionally, we explored potential pathways related to risk scores, providing new insights into the molecular mechanisms by which CAFs promote BRCA progression.

## 2 Materials and methods

### 2.1 Datasets and data preprocessing

We obtained normalized expression data and clinical information in GSE7390 (n = 198), GSE20685 (n = 327), and GSE25065 (n = 198) were obtained from GEO database. The probes were mapped using the corresponding annotation platforms. If a gene symbol was recorded with multiple probes, the average value was used as its expression level. GSE7390 and GSE20685 were merged and normalized using the Combat function of the “sva” package.

### 2.2 CAFs infiltration and stromal score calculation

CAFs infiltration and stromal scores were calculated using EPIC [21], xCell [22], MCP counter [23], TIDE [24], and ESTIMATE algorithms [25].

### 2.3 WGCNA

We used the WGCNA package to screen hub genes that were significantly related to CAFs scores. The genes in the module were screened as potential CAFs-related genes when MM was >0.6 and GS was >0.6. Obtain the hub module after adjacency matrix was clustered and the soft threshold.

### 2.4 Enrichment analysis

The Gene Ontology (GO), the Kyoto Encyclopedia of Genes and Genomes (KEGG), and Gene Set Enrichment Analysis (GSEA) were performed using the “clusterProfiler” and “GSVA” packages.

### 2.5 Construction and validation of the prognostic model

The combined cohort of GSE7390 and GSE20685 was used to construct the CAFs risk model, and GSE25065 served as a validation cohort. Prognostic CAFs hub genes were determined by performing a univariate Cox regression analysis and the CAFs risk model was constructed by Lasso regression analysis based on genes with P < 0.03. Kaplan–Meier survival curves were used to visualize survival differences in the high- and low-risk groups of patients with BRCA. Moreover, the CAFs risk model was validated in the GSE25065 cohort.

### 2.6 Prediction of chemotherapy sensitivity and immunotherapy responses

The half-maximal inhibitory concentrations of common drugs administered to each patient with BRCA were studied using the “pRRophetic” package [26]. The online TIDE algorithm was used to predict response to immune checkpoint blocking treatment and evaluate the predictive power of CAFs risk signature.

### 2.7 Expression characteristics of a CAFs gene signature

We verified the mRNA expression of four genes (COL5A3, FN1, POSTN, and RARRES2) in CAFs and BRCA cell lines using the Cancer Cell Line Encyclopedia (CCLE) database [27]. The protein levels of the four genes in BRCA tissues were obtained using the Human Protein Atlas (HPA) database [28]. Additionally, the TISCH database was used for validation at the single-cell level [29].

### 2.8 Cell experiment validation

Primary CAFs were isolated from breast tumor tissues that were cut into pieces by digesting with collagenase II and hyaluronidase for 15 min. For Western Blotting, electrophoresis and membrane transfer were performed on the extract relevant proteins. After incubation with the primary and secondary antibodies, protein bands were detected using chemiluminescence reagents. The cell lines, wound healing and invasion assays were performed as previously described [30].

### 2.9 Statistical analysis

Statistical analyses were performed using the R software. Wilcoxon test was suitable for paired comparisons, and the Kaplan–Meier curve was plotted to analyze survival differences in the two groups. P-value <0.05 was considered statistically significant.

## 3 Results

### 3.1 Relationship between CAFs infiltration scores and prognosis

The flowchart of the study is presented in Fig. 1. Multiple predictions of CAFs infiltration scores suggested that in the combined cohort of GSE7390 and GSE20685, high CAFs infiltration scores (MCP counter, EPIC, and TIDE algorithms) were significantly correlated to the poor prognosis of patients with BRCA (Fig. 2A). In the GSE25065 cohort, higher CAFs infiltration (MCP counter) and stromal scores were significantly associated with a poor prognosis. We considered the CAFs abundance calculated by the MCP counter algorithm for subsequent analysis, whereas we used the remaining algorithms for validation (Fig. 2B).

**Fig. 1:**
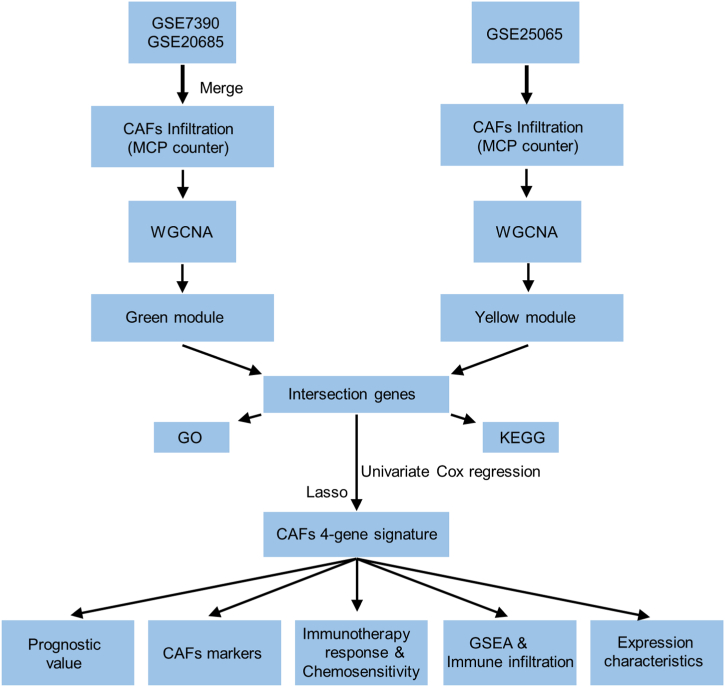
Flowchart depicting the flow of the present research.

**Fig. 2:**
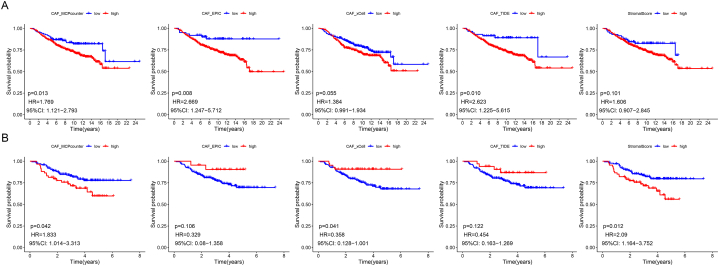
Correlation of CAFs infiltration and stromal score with the prognosis in combined cohorts (**A**) and GSE25065 cohorts (**B**).

### 3.2 Co-expression network of CAFs scores

**Fig. 3:**
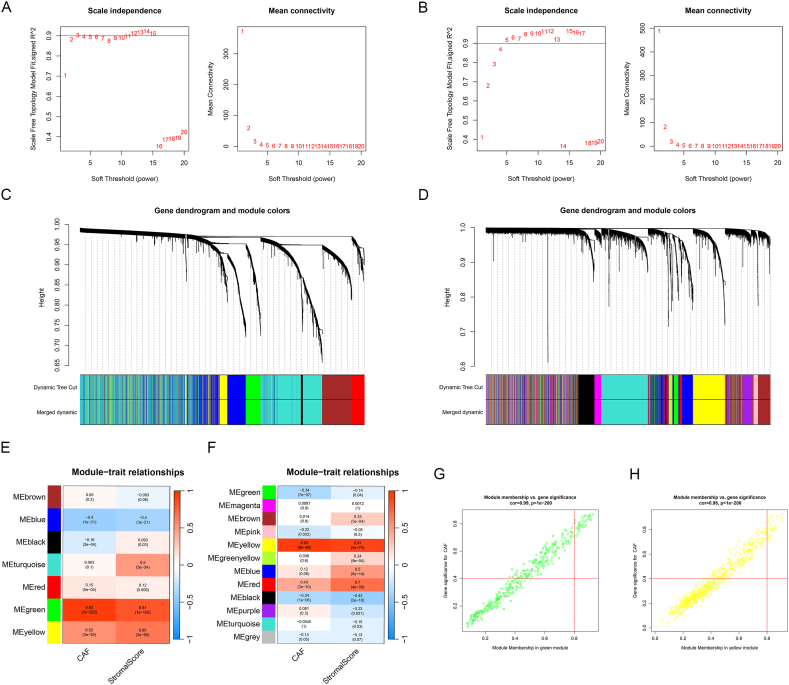
WGCNA in the combined cohorts and GSE25065 cohorts. (**A-B**) The soft thresholding power of the combined cohorts (**A**) and GSE25065 cohorts (**B**). (**C-D**) Clustering dendrograms of the combined cohorts (**C**) and GSE25065 cohorts (**D**). (**E-F)** Heatmap displaying the correlation between module characteristic genes and CAFs infiltration score and stromal score in the combined cohorts (**E**) and GSE25065 cohorts (**F**). (**G-H**) Scatter diagram depicting the relationship between MM and GS in green (**G**) and yellow modules (**H**). (For interpretation of the references to color in this figure legend, the reader is referred to the Web version of this article.)

The WGCNA showed that the soft threshold power of the combined cohort was three when seven co-expression models were clustered. The correlation between green modules and CAFs infiltration scores was strongest (Fig. 3A–C, and E). When 12 co-expression models were clustered, the soft threshold power was five and the yellow module showed the strongest correlation with CAFs infiltration scores in the GSE25065 cohort (Fig. 3B–D, and F). A total of 81 and 108 genes were integrated into the green and yellow modules, respectively. Fig. 3G and H shows that the correlation between MM and GS was 0.99 and 0.98, respectively.

### 3.3 Functional analysis of hub genes

**Fig. 4:**
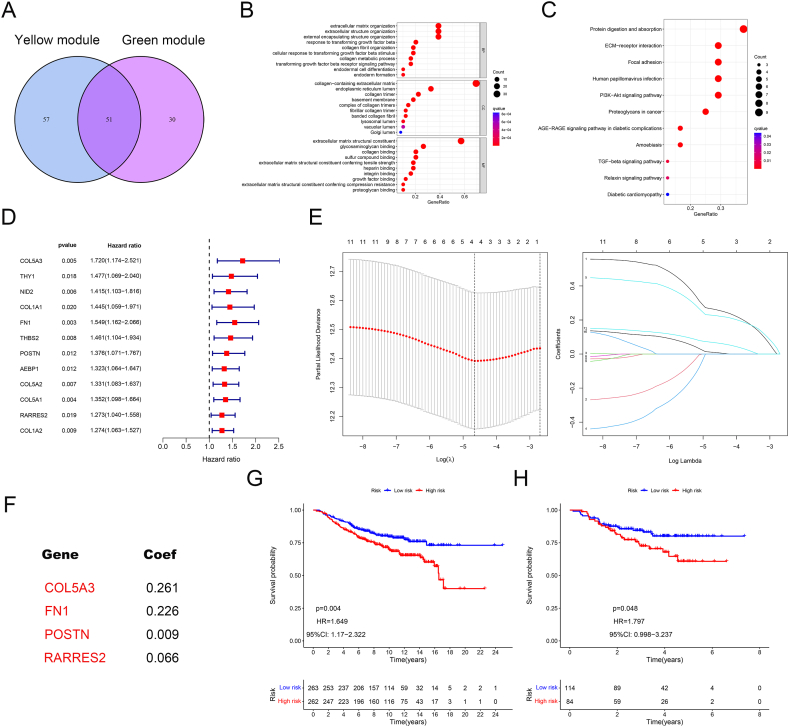
Functional analysis and establishment of CAFs gene signature. (**A**) The intersection gene of the green and yellow modules. (**B–C**) GO and KEGG analyses of the intersection genes. (**D-E**) Univariate Cox and Lasso Regression Analysis of the intersecting genes. (**F**) The establishment of the CAFs risk model. (**G-H**) Survival analyses of the combined cohorts (**G**) and GSE25065 cohorts (**H**). (For interpretation of the references to color in this figure legend, the reader is referred to the Web version of this article.)

A total of 51 intersection genes in the yellow and green modules were visualized using a Venn diagram (Fig. 4A). The results of the GO analysis indicated that the main enriched biological process terms were extracellular matrix organization, extracellular structure organization, and external encapsulating structure organization. The main enriched cellular component and molecular function terms were collagen-containing extracellular matrix and extracellular matrix structural constituent, respectively (Fig. 4B). The KEGG pathways that showed significant enrichment were protein digestion and absorption, ECM-receptor interaction, focal adhesion, human papillomavirus infection, and PI3K/AKT signaling (Fig. 4C).

### 3.4 Construction of the CAFs risk model

The combined cohorts of GSE7390 and GSE20685 served as a training cohort, whereas the GSE25065 served as a validation cohort. A total of 12 prognostic-related genes were selected by performing univariate Cox regression analysis (Fig. 4D). Subsequently, we performed Lasso Cox regression analysis and identified four genes for constructing the CAFs risk model. The scores were calculated as follows: CAFs risk score = COL5A3 expression × 0.261 + FN1 expression × 0.226 + POSTN expression × 0.009 + RARRES2 expression × 0.066 (Fig. 4E–F). Fig. 4G and H showed that high-risk patients predicted a poor prognosis in the training and validation cohorts.

### 3.5 Relationship between the CAFs gene signature and CAFs infiltration scores and CAFs markers

**Fig. 5:**
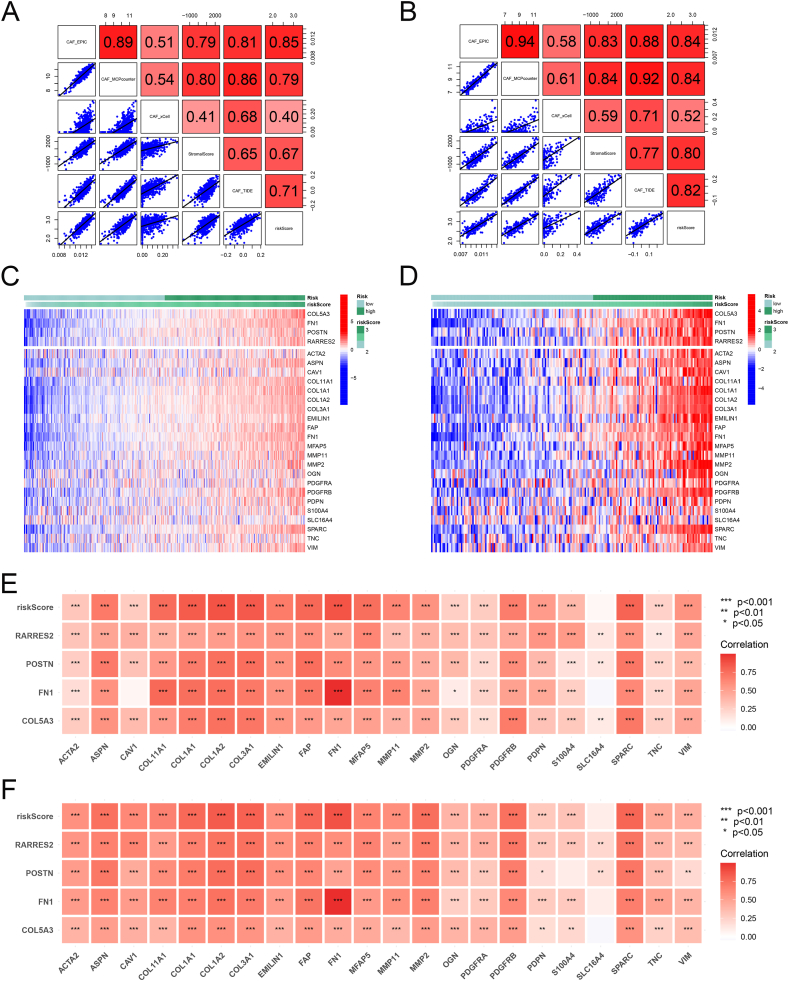
CAFs gene signature correlated with CAFs infiltration and CAFs markers. (**A-B**) Correlation between the CAFs risk scores with CAFs infiltration and stromal score in the combined cohorts (**A**) and GSE25065 cohorts (**B**). (**C-D**) Heatmap of CAFs marker expression in different risk groups in the combined cohorts (**C**) and GSE25065 cohorts (**D**). (**E-F**) Heatmap displaying the correlation between four hub genes and CAFs marker genes in the combined cohorts (**E**) and GSE25065 cohorts (**F**).

We found a significant correlation between risk scores and CAFs infiltration and stromal scores in both the combined and GSE25065 cohorts (Fig. 5A–B). Additionally, 22 clinical CAFs marker genes were highly expressed in the high-risk group (Fig. 5C–D). Furthermore, a strong positive correlation between the levels of clinical CAFs markers and the expression of the four signature genes was observed (Fig. 5E–F).

### 3.6 Chemotherapy and immunotherapy responses of the CAFs gene signature

**Fig. 6:**
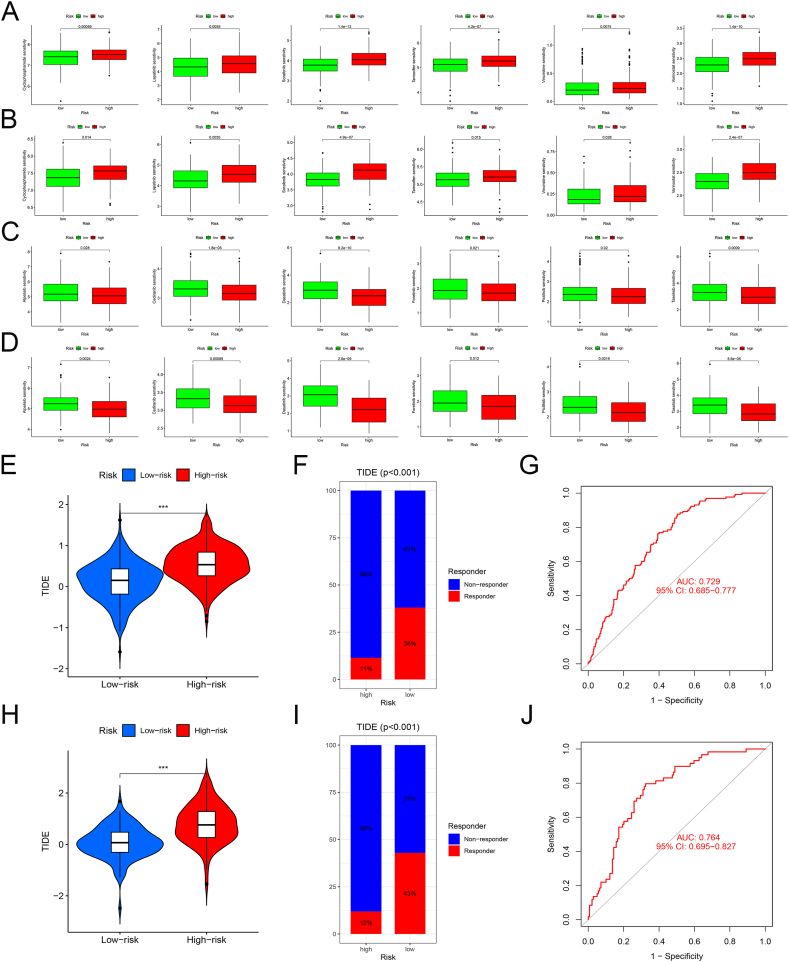
Responses of the patients in the high- and low-risk groups to chemotherapy and immunotherapy. (**A-B**) Drugs sensitive to chemotherapy in the low-risk group of the combined cohorts (**A**) and GSE25065 cohorts (**B**). (**C-D**) Drugs sensitive to chemotherapy in the high-risk group of the combined cohorts (**C**) and GSE25065 cohorts (**D**). (**E, H**) High-risk patients with higher TIDE scores in the combined cohorts (**E**) and GSE25065 cohorts (**H**). (**F, I**) High-risk patients with a higher proportion of non-responders in the combined cohorts (**F**) and GSE25065 cohorts (**I**). (**G, J**) The ROC curve and area under the curve of CAFs risk score predicted immunotherapy response in the combined cohorts (**G**) and GSE25065 cohorts (**J**).

Chemotherapy is an important means of treating BRCA. Patients in the low-risk group were sensitive to Cyclophosphamide, Lapatinib, Sorafenib, Tamoxifen, Vincristine, and Vorinostat, whereas those in the high-risk group were sensitive to Alpelisib, Cediranib, Dasatinib, Foretinib, Pictilisib, and Taselisib (Fig. 6A–B). The positive effects of immunotherapy have been observed in various cancers, providing better treatment options for patients. We used the TIDE algorithm to assess the potential of CAFs risk scores as a predictive marker for immunotherapy. The proportion of non-responders to immunotherapy was higher in the high-risk group than that in the low-risk group in the combined and GSE25065 cohorts (Fig. 6C–D). This indicated that CAFs models could predict immune treatment responses to a certain extent.

### 3.7 GSEA of CAFs gene signature

**Fig. 7:**
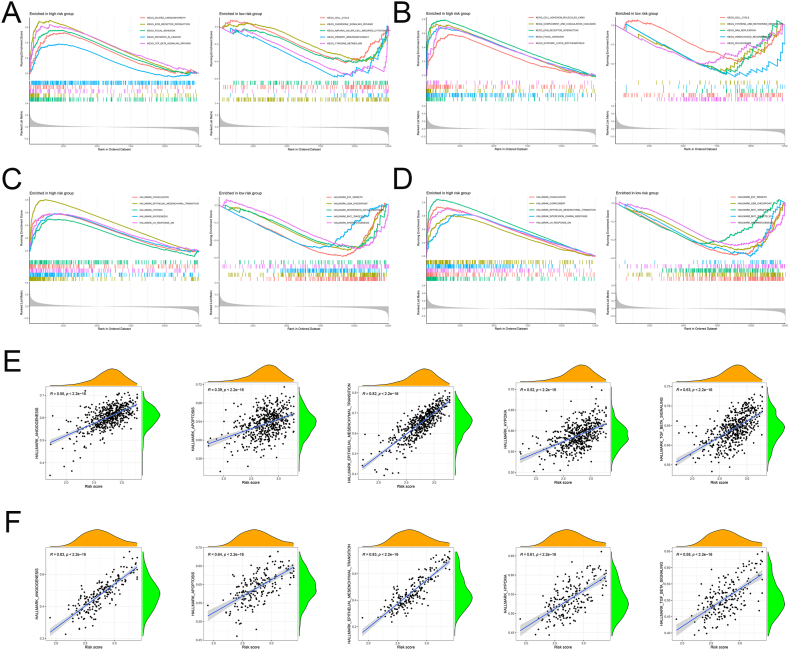
Biological pathways of the different groups. (**A-B**) The GSEA analysis for the KEGG pathway in the combined cohorts (**A**) and GSE25065 cohorts (**B**). (**C-D**) The GSEA analysis for the hallmark in the combined cohorts (**C**) and GSE25065 cohorts (**D**). (**E-F**) Correlational analysis between CAFs risk scores with angiogenesis, apoptosis, EMT, hypoxia, and TGF-β signaling in the combined cohorts (**E**) and GSE25065 cohorts (**F**).

To further clarify the molecular function of CAFs gene signature, we performed GSEA and the results showed that the high-risk groups of both cohorts were significantly enriched in ECM receptor interactions, Focal adhesion, Coagulation, epithelial-mesenchymal transition (EMT), and UV response Dn, which consistent with the invasive behavior of CAFs (Fig. 7A–D). Furthermore, CAFs risk scores were strongly positively associated with angiogenesis, apoptosis, EMT, hypoxia, and TGF-β signaling (Fig. 7E–F).

### 3.8 Relationship between CAFs gene signature and TME

**Fig. 8:**
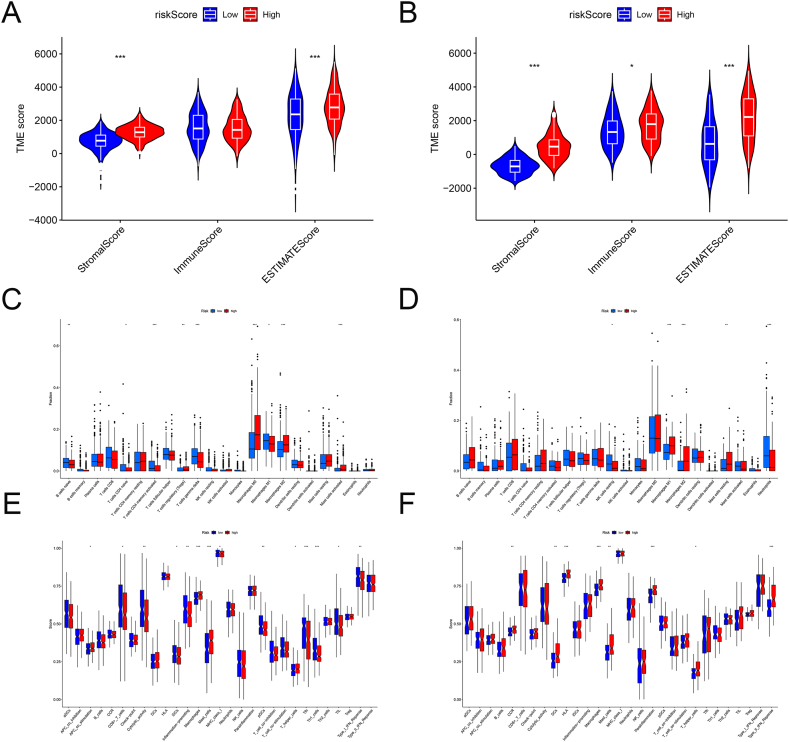
Correlation analysis between CAFs risk score and TME. (**A-B**) Relationship between the CAFs risk scores and stromal, immune, and ESTIMATE scores in the combined cohorts (**A**) and GSE25065 cohorts (**B**). (**C-D**) Immune cell infiltration differences between the different risk groups of the combined cohorts (**C**) and GSE25065 cohorts (**D**). (**E-F**) Differences in the immune functions between different risk groups of the combined cohorts (**E**) and GSE25065 cohorts (**F**).

In the combined cohort, CAFs risk scores were significantly positively correlated to stromal and ESTIMATE scores, whereas in the GSE250265 cohort, they were significantly positively correlated to stromal, immune, and ESTIMATE scores (Fig. 8A–B). Using CIBERSORT, we found that patients with high CAFs risk scores showed a higher proportion of activated regulatory T cells, M0 and M2 macrophages, and mast cells, whereas patients with low CAFs risk scores showed a higher proportion of activated naive B cells, CD4^+^ naive T cells, CD4^+^ memory T cells, gamma delta T cells, and M1 macrophages (Fig. 8C–D). ssGSEA showed that patients with high CAFs risk scores were enriched in antigen-presenting cell co-stimulation, macrophages, mast cells, and T-helper (Th) cells, whereas patients with low CAFs risk scores were enriched in CD8^+^ T cells, cytological activity, immature dendritic cells (iDCs), inflammation promotion, major histocompatibility complex class I, plasmacytoid DCs, T follicular helper cells, Th1 cells, tumor-infiltrating lymphocytes (TILs), and type I interferon responses (Fig. 8E–F). It was worth noting that groups with high CAFs risk scores in both cohorts were associated with M2 macrophages. Furthermore, despite the results of the ssGSEA analysis differed between the two cohorts, the high-risk groups in both cohorts were associated with macrophages, mast cells, and Th cells. These findings indicated that CAFs in high-risk groups might form an immunosuppressive TME by inducing the infiltration of immunosuppressive cells.

### 3.9 Expression characteristic of CAFs gene signature

**Fig. 9:**
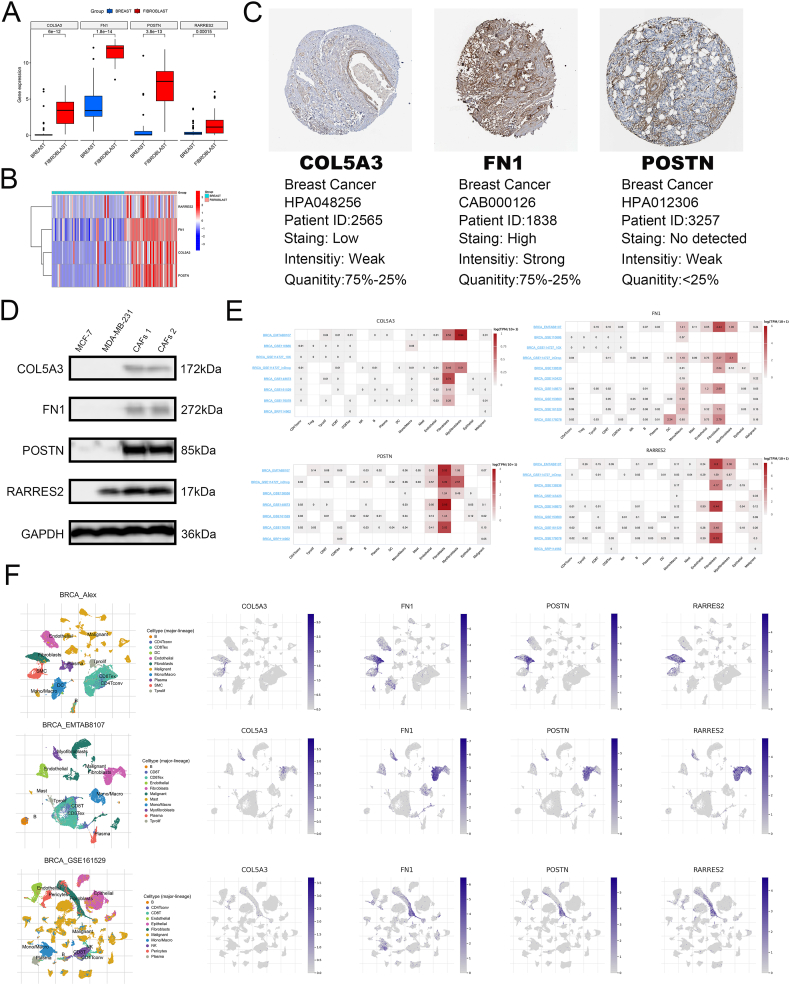
Expression characteristics of 4 genes. (**A-B)** The expression of 4 genes in CAFs and BRCA cell lines. (**C)** IHC images in the HPA database. (**D)** Western blotting of 4 genes. (**E-F)** Expression of 4 genes in TME from the TISCH database.

**Fig. 10:**
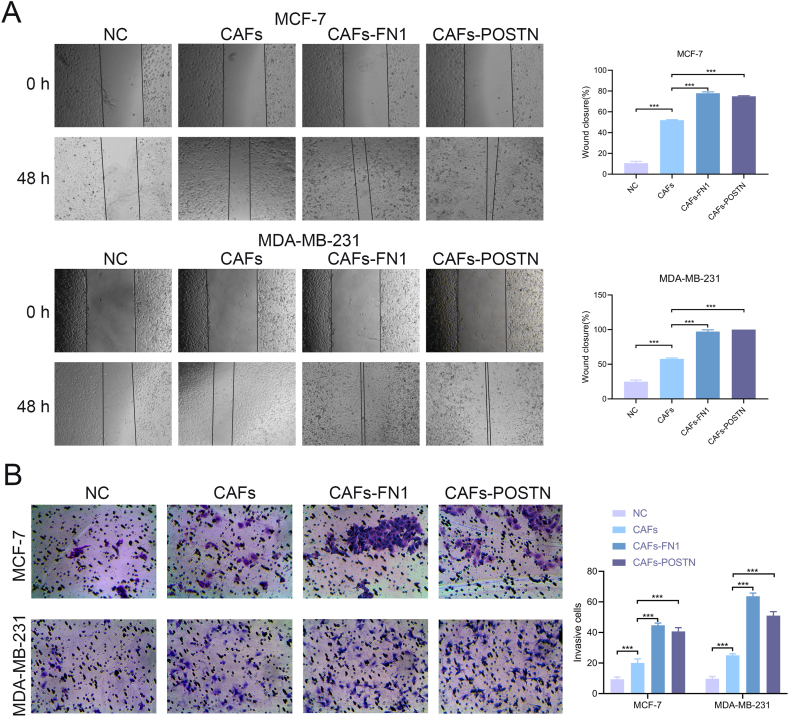
In vitro validation. (**A-B)** CAFs overexpressing FN1 and POSTN significantly promoted the wound healing and invasion ability of tumor cells.

Using the CCLE database, we observed that the expression of COL5A3, FN1, POSTN, and RARRES2 was higher in fibroblast cell lines than in BRCA cell lines (Fig. 9A–B). Additionally, the HPA database showed that FN1 was deeply stained, whereas COL5A3 and POSTN were weakly stained in the stroma of BRCA (Fig. 9C). Unfortunately, the HPA database did not have immunohistochemistry images for RARRES2. Western blotting showed that the expression of the four hub genes was higher in primary CAFs than in cancer cell lines (Fig. 9D). Furthermore, the TISCH database showed that COL5A3, FN1, POSTN, and RARRES2 were significantly overexpressed in fibroblasts (Fig. 9E–F). Through cell experiments, we found hat overexpression of FN1 and POSTN in CAFs significantly promoted the wound healing and invasion ability of tumor cells (Fig. 10A–B). In summary, these four genes may be specific markers for CAFs.

## 4 Discussion

Tumors are highly heterogeneous and contain different cellular and peripheral components that intricately interact with each other [31]. CAFs play a major role in all stages of tumor development, growth, invasion, and distant metastasis. In the present study, higher MCP counter scores were associated with poorer prognosis. Thus, based on univariate Cox and LASSO regression analyses, we constructed and validated a four-gene (COL5A3, FN1, POSTN, and RARRES2) prognostic CAFs model, which could accurately assess the level of CAFs infiltration and predict immune and chemotherapy responses. The identified prognostic markers of CAFs will help enhance the overall understanding of CAFs and develop new stromal therapies for targeting CAFs.

Compared to normal fibroblasts, CAFs display a distinct activated phenotype and play a crucial role in tumor progression [32]. Therefore, it is imperative to update clinical strategies for targeting CAFs in order to achieve more effective tumor treatment. Here, we have developed a 4-gene CAFs risk model that accurately predicts patient prognosis and their response to immunotherapy and chemotherapy, thereby providing a personalized treatment strategy for BRCA patients. We found that patients with a high CAFs risk score were associated with poor prognosis and less responsive to immunotherapy, but more sensitive to Alpelisib, Cediranib, Dasatinib, Foretinib, Pictilisib, and Taselisib. Thus, in clinical practice, administering neoadjuvant chemotherapy with highly sensitive drugs to patients with a high CAFs risk score may prove advantageous, as it can reduce tumor volume and enhance the efficacy of surgical resection, ultimately improving patient survival. Additionally, by analyzing gene expression in BRCA patients to obtain the CAFs risk score, we can predict their response to immunotherapy and chemotherapy. Based on these predictions, doctors can make informed decisions on whether to administer immunotherapy drugs, such as immune checkpoint inhibitors, or select effective chemotherapy drugs, thus developing personalized treatment plans based on individual patient differences.

Our study revealed a significant enrichment of ECM-related functions in the intersecting genes. ECM synthesis and reshaping is the most unique function of CAFs. ECM is mainly secreted by activated CAFs, providing key signals to maintain tissue scaffold structures and organ homeostasis, as well as control tumor growth, recurrence, metastasis, and drug resistance. Specifically, ECM forms a protective barrier around the cancer nest, blocking the penetration of chemicals, immune drugs and host immune cells. Furthermore, it promotes tumor cell migration and invasion by providing an environment conducive to tumor cell interactions with cytokines [33].

A strong positive correlation was observed between CAFs risk scores and angiogenesis, EMT, and hypoxia, which might be the important molecular mechanisms for immunotherapy resistance and a poor prognosis in patients with high-risk scores. Previous studies have demonstrated that VEGF-mediated angiogenesis promotes the infiltration of immunosuppressive cells, such as Tregs and myelogenous inhibitory cells, ultimately leading to the formation of an immunosuppressive microenvironment [[34], [35], [36]]. Additionally, various soluble cytokines secreted by CAFs promoted EMT via TGF-β signal pathways, promoting the malignant behavior of tumor cells, including proliferation, migration, and invasion, and leading to resistance to chemotherapy and immunotherapy [37,38]. Furthermore, hypoxia inhibited the tumor-suppressive function of various immune cells, thereby contributing to immunotherapy resistance [39,40]. It is worth noting that hypoxia can trigger angiogenesis and EMT, further exacerbating immunosuppression [[41], [42], [43], [44]]. The CIBERSORT analysis showed that high CAFs risk scores were positively correlated to Tregs and M2 macrophage polarization, and the ssGSEA analysis showed that high CAFs risk scores were negatively correlated to CD8^+^ T cells and TILs, which might be the mechanism leading to immunotherapy resistance in patients with high-risk scores. However, this finding needs further validation in vitro and *in vivo*.

Numerous studies have reported the tumorigenic effects of the four genes in the CAFs signature on cancer, particularly FN1. FN1 is highly expressed in various cancers, including gastric, colorectal, and thyroid cancers and BRCA, and is correlated to a poor prognosis [[45], [46], [47], [48]]. Furthermore, the downregulation of FN1 inhibits the proliferation, migration, and invasion of colorectal cancer cells [49]. COL5A3 is correlated to BRCA metastasis to the brain and the tumor stemness of head and neck squamous cell carcinoma [50,51]. POSTN is highly expressed in stromal components, especially fibroblasts, of ovarian cancer. By activating the PI3K/AKT pathway and inducing EMT, CAFs-derived POSTN promotes the migration and invasion of ovarian cancer cells, thus leading to a poor prognosis [52]. RARRES2, a fat factor, is the key participant in initiating early immune responses, which facilitates the proliferation and migration of tumor cells by upregulating the PD-L1 gene [53]. However, the potential role of these four genes in BRCA is still poorly understood and requires further verification.

Currently, there are several effective prognostic models for BRCA [54,55]. However, these bioinformatics-based models still have some potential limitations. Firstly, the predictive results of these models still need to be validated in clinical practice. Although our model performed well in the training and validation cohorts in our study, further validation is still required in real clinical settings. Secondly, the predictive ability of this model may be influenced by other unconsidered factors, such as patient age, pathological type, drugs, and dosage. Therefore, in practical applications, it is necessary to consider these factors comprehensively to formulate the optimal treatment plan.

## 5 Conclusions

We identified a new prognostic biomarker for CAFs using a bioinformatics tool. The constructed four-gene CAFs risk model provides a new strategy for developing personalized treatment plans. However, further molecular and animal experiments are necessary to verify the biological role of the CAFs signature biomarkers in BRCA.

## Ethics statement

This study was approved by the Ethics Committee of the Seventh Medical Center of the PLA General Hospital (2023–37). All the patients signed an informed consent form prior to the surgery and complied with all regulations.

## Funding

This research did not receive any specific grant from funding agencies in the public, commercial, or not-for-profit sectors.

## Data availability statement

The datasets analyzed during the current study are available in the GEO data portal (https://www.ncbi.nlm.nih.gov/geo/). All the remaining data are available from the authors upon reasonable request.

## CRediT authorship contribution statement

**Jin Song:** Writing – original draft, Conceptualization. **Huifeng Liao:** Writing – original draft, Conceptualization. **Huayan Li:** Methodology. **Hongye Chen:** Methodology. **Huiyan Si:** Methodology. **Jiandong Wang:** Supervision. **Xue Bai:** Supervision.

## Declaration of competing interest

The authors declare that they have no known competing financial interests or personal relationships that could have appeared to influence the work reported in this paper.

## Figures and Tables

**figs1:**
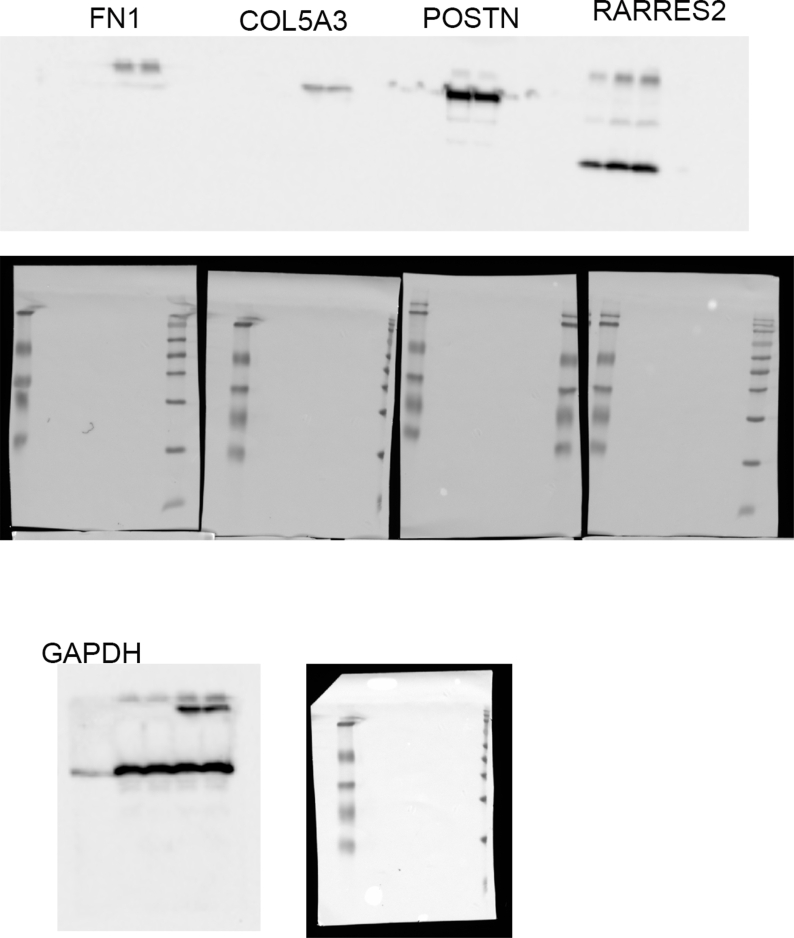


## References

[1] Loibl S., Poortmans P., Morrow M., Denkert C., Curigliano G. (2021). Breast cancer. Lancet.

[2] Musgrove E.A., Sutherland R.L. (2009). Biological determinants of endocrine resistance in breast cancer. Nat. Rev. Cancer.

[3] Liang H., Lu Q., Yang J., Yu G. (2023).

[4] Wang X., Zhang H., Chen X. (2019). Drug resistance and combating drug resistance in cancer. Cancer Drug Resist.

[5] Biffi G., Tuveson D.A. (2021). Diversity and biology of cancer-associated fibroblasts. Physiol. Rev..

[6] Tang J., He J., Guo H. (2023).

[7] Li C., Teixeira A.F., Zhu H.J., Ten Dijke P. (2021). Cancer associated-fibroblast-derived exosomes in cancer progression. Mol. Cancer.

[8] Kobayashi H., Enomoto A., Woods S.L. (2019). Cancer-associated fibroblasts in gastrointestinal cancer. Nat. Rev. Gastroenterol. Hepatol..

[9] Zheng S., Liang J.Y., Tang Y. (2023). Dissecting the role of cancer-associated fibroblast-derived biglycan as a potential therapeutic target in immunotherapy resistance: a tumor bulk and single-cell transcriptomic study. Clin. Transl. Med..

[10] Qi R., Bai Y., Li K. (2023). Cancer-associated fibroblasts suppress ferroptosis and induce gemcitabine resistance in pancreatic cancer cells by secreting exosome-derived ACSL4-targeting miRNAs. Drug Resist. Updates.

[11] Czekay R.P., Cheon D.J., Samarakoon R., Kutz S.M., Higgins P.J. (2022). Cancer-associated fibroblasts: mechanisms of tumor progression and novel therapeutic targets. Cancers.

[12] Harper J., Sainson R.C. (2014). Regulation of the anti-tumour immune response by cancer-associated fibroblasts. Semin. Cancer Biol..

[13] Jain R.K., Lahdenranta J., Fukumura D. (2008). Targeting PDGF signaling in carcinoma-associated fibroblasts controls cervical cancer in mouse model. PLoS Med..

[14] Mao X., Xu J., Wang W. (2021). Crosstalk between cancer-associated fibroblasts and immune cells in the tumor microenvironment: new findings and future perspectives. Mol. Cancer.

[15] Takahashi H., Sakakura K., Kudo T. (2017). Cancer-associated fibroblasts promote an immunosuppressive microenvironment through the induction and accumulation of protumoral macrophages. Oncotarget.

[16] Unterleuthner D., Neuhold P., Schwarz K. (2020). Cancer-associated fibroblast-derived WNT2 increases tumor angiogenesis in colon cancer. Angiogenesis.

[17] Langfelder P., Horvath S. (2008). WGCNA: an R package for weighted correlation network analysis. BMC Bioinf..

[18] Zheng H., Liu H., Li H., Dou W., Wang X. (2021). Weighted gene Co-expression network analysis identifies a cancer-associated fibroblast signature for predicting prognosis and therapeutic responses in gastric cancer. Front. Mol. Biosci..

[19] Zhao Z., Mak T.K., Shi Y. (2023). Integrative analysis of cancer-associated fibroblast signature in gastric cancer. Heliyon.

[20] Feng S., Xu Y., Dai Z. (2022). Integrative analysis from multicenter studies identifies a WGCNA-derived cancer-associated fibroblast signature for ovarian cancer. Front. Immunol..

[21] Racle J., de Jonge K., Baumgaertner P., Speiser D.E., Gfeller D. (2017). Simultaneous enumeration of cancer and immune cell types from bulk tumor gene expression data. Elife.

[22] Aran D., Hu Z., Butte A.J. (2017). xCell: digitally portraying the tissue cellular heterogeneity landscape. Genome Biol..

[23] Becht E., Giraldo N.A., Lacroix L. (2016). Estimating the population abundance of tissue-infiltrating immune and stromal cell populations using gene expression. Genome Biol..

[24] Jiang P., Gu S., Pan D. (2018). Signatures of T cell dysfunction and exclusion predict cancer immunotherapy response. Nat. Med..

[25] Yoshihara K., Shahmoradgoli M., Martínez E. (2013). Inferring tumour purity and stromal and immune cell admixture from expression data. Nat. Commun..

[26] Geeleher P., Cox N., Huang R.S. (2014). pRRophetic: an R package for prediction of clinical chemotherapeutic response from tumor gene expression levels. PLoS One.

[27] Ghandi M., Huang F.W., Jané-Valbuena J. (2019). Next-generation characterization of the cancer cell line Encyclopedia. Nature.

[28] Uhlén M., Fagerberg L., Hallström B.M. (2015). Proteomics. Tissue-based map of the human proteome. Science.

[29] Sun D., Wang J., Han Y. (2021). TISCH: a comprehensive web resource enabling interactive single-cell transcriptome visualization of tumor microenvironment. Nucleic Acids Res..

[30] Liao H., Li H., Dong J. (2023). Melatonin blunts the tumor-promoting effect of cancer-associated fibroblasts by reducing IL-8 expression and reversing epithelial-mesenchymal transition. Int. Immunopharm..

[31] Meacham C.E., Morrison S.J. (2013). Tumour heterogeneity and cancer cell plasticity. Nature.

[32] Hu D., Zhuo W., Gong P. (2023). Biological differences between normal and cancer-associated fibroblasts in breast cancer. Heliyon.

[33] Kalluri R. (2016). The biology and function of fibroblasts in cancer. Nat. Rev. Cancer.

[34] Wada J., Suzuki H., Fuchino R. (2009). The contribution of vascular endothelial growth factor to the induction of regulatory T-cells in malignant effusions. Anticancer Res..

[35] Rahma O.E., Hodi F.S. (2019). The intersection between tumor angiogenesis and immune suppression. Clin. Cancer Res..

[36] Yang J., Yan J., Liu B. (2018). Targeting VEGF/VEGFR to modulate antitumor immunity. Front. Immunol..

[37] Hao Y., Baker D., Ten Dijke P. (2019). TGF-β-Mediated epithelial-mesenchymal transition and cancer metastasis. Int. J. Mol. Sci..

[38] Yang M., Li D., Jiang Z. (2022). TGF-β-Induced FLRT3 attenuation is essential for cancer-associated fibroblast-mediated epithelial-mesenchymal transition in colorectal cancer. Mol. Cancer Res..

[39] Kopecka J., Salaroglio I.C., Perez-Ruiz E. (2021). Hypoxia as a driver of resistance to immunotherapy. Drug Resist. Updates.

[40] Wang B., Zhao Q., Zhang Y. (2021). Targeting hypoxia in the tumor microenvironment: a potential strategy to improve cancer immunotherapy. J. Exp. Clin. Cancer Res..

[41] Wicks E.E., Semenza G.L. (2022). Hypoxia-inducible factors: cancer progression and clinical translation. J. Clin. Invest..

[42] Germain S., Monnot C., Muller L., Eichmann A. (2010). Hypoxia-driven angiogenesis: role of tip cells and extracellular matrix scaffolding. Curr. Opin. Hematol..

[43] Lin Y.T., Wu K.J. (2020). Epigenetic regulation of epithelial-mesenchymal transition: focusing on hypoxia and TGF-β signaling. J. Biomed. Sci..

[44] Nushtaeva A., Ermakov M., Abdurakhmanova M. (2023). Pulsed hypoxia" gradually reprograms breast cancer fibroblasts into pro-tumorigenic cells via mesenchymal-epithelial transition. Int. J. Mol. Sci..

[45] Wang H., Zhang J., Li H. (2022). FN1 is a prognostic biomarker and correlated with immune infiltrates in gastric cancers. Front. Oncol..

[46] Wang J., Li R., Li M., Wang C. (2021). Fibronectin and colorectal cancer: signaling pathways and clinical implications. J. Recept. Signal Transduct. Res..

[47] Geng Q.S., Huang T., Li L.F. (2021). Over-expression and prognostic significance of FN1, correlating with immune infiltrates in thyroid cancer. Front. Med..

[48] Zhang X.X., Luo J.H., Wu L.Q. (2022). FN1 overexpression is correlated with unfavorable prognosis and immune infiltrates in breast cancer. Front. Genet..

[49] Cai X., Liu C., Zhang T.N. (2018). Down-regulation of FN1 inhibits colorectal carcinogenesis by suppressing proliferation, migration, and invasion. J. Cell. Biochem..

[50] Zhang L., Wang L., Yang H., Li C., Fang C. (2021). Identification of potential genes related to breast cancer brain metastasis in breast cancer patients. Biosci. Rep..

[51] Wu Z.H., Li C., Zhang Y.J., Zhou W. (2022). Identification of a cancer stem cells signature of head and neck squamous cell carcinoma. Front. Genet..

[52] Yue H., Li W., Chen R. (2021). Stromal POSTN induced by TGF-β1 facilitates the migration and invasion of ovarian cancer. Gynecol. Oncol..

[53] Gao C., Shi J., Zhang J., Li Y., Zhang Y. (2022). Chemerin promotes proliferation and migration of ovarian cancer cells by upregulating expression of PD-L1. J. Zhejiang Univ. - Sci. B.

[54] Zhou L., Rueda M., Alkhateeb A. (2022). Classification of breast cancer nottingham prognostic index using high-dimensional embedding and residual neural network. Cancers.

[55] Tabl A.A., Alkhateeb A., ElMaraghy W., Rueda L., Ngom A. (2019). A machine learning approach for identifying gene biomarkers guiding the treatment of breast cancer. Front. Genet..

